# Un cas rare de galactocèle géant associé à un prolactinome

**DOI:** 10.11604/pamj.2017.27.97.12484

**Published:** 2017-06-08

**Authors:** Mariam Tayae, Jihad Jamor

**Affiliations:** 1 Service de Gynécologie Obstétrique II, CHU Hassan II, Fès, Maroc

**Keywords:** Galactocèle, prolactinome, sein, Galactocele, prolactinoma, breast

## Abstract

Le galactocèle est une forme kystique rare, c'est une lésion bénigne du sein, apparaissant quand un canal mammaire est obstrué et très rempli de lait. On le rencontre généralement chez les femmes en post-partum, soit allaitant ou non. Il n'y a que quelques cas signalés qui ne sont pas liées à la lactation, comme on le voit chez les femmes ménopausées ou chez les hommes; tant plus, que la relation à la surproduction de prolactine, un facteur de croissance de l'épithélium mammaire n'est pas très bien défini à ce moment. Nous présentons un tel cas inhabituel d'une patiente âgée de 30 ans qui n'a pas d'antécédents d'accouchement ou l'avortement. Elle est suivie en endocrinologie pour micro adénome hypophysaire mise sous Cabergoline* qu'elle a arrêté d'elle-même pendant un an. Même compte tenu de la rareté de l'association, il est important de souligner le rôle hormonal dans l'évolution de l'anatomie du sein.

## Abstract (en)

Galactocele is a rare cystic formation, a benign breast lesion, occurring when breast duct is blocked and engorged. It generally affects postpartum women, either breastfeeding or not. Only a few cases have been reported in the literature and they were not related to lactation, as in the case of postmenopausal women or of men; moreover, their relationship to the overproduction of prolactin, a growth factor stimulating mammary epithelial cells, is not very well defined at this time. We here report the unusual case of a 30-year old patient with no personal history of childbirth or abortion. She was treated in the Division of Endocrinology for pituitary microadenoma with Cabergoline, that she stopped for 1 year. Even taking into account this rare association, it is important to emphasize the role of hormones in the progression of breast anatomy.

## Introduction

Le galactocèle est une lésion kystique bénigne du sein, contenant du lait ou un liquide laiteux, il est dû à l'accumulation de liquide de sécrétion suite à l'obstruction d'un canal galactophore. La plupart des cas sont rapportés à la grossesse, en particulier à la période d'allaitement. Une partie d'entre eux est révélé des années après l'allaitement. Par ailleurs, le kyste est décrit dans certains cas inhabituels lorsque l'obstruction est causée par un obstacle mécanique comme un papillome ou implants mammaires. Il n'y a que quelques cas de galactocèle chez les hommes probablement causés par les crises génitales néonatales. L'association avec l'hyperprolactinémie est soutenue par un cas de galactocèle chez un homme avec macro-prolactinome, et un autre chez une femme de 37 ans avec micro-prolactinome, avec une rémission après un traitement approprié du prolactinome [1]. Notre cas est également lié à des niveaux élevés de prolactine chez une patiente de 30 ans.

## Patient et observation

**Figure 1:**
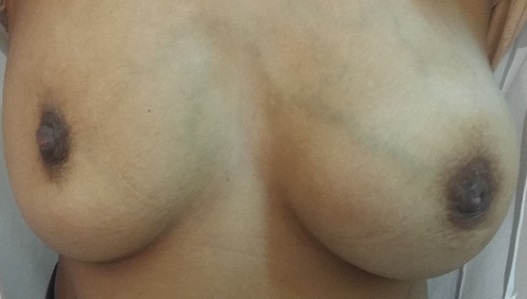
Masse de 12cm/7 prenant tout le sein gauche

**Figure 2:**
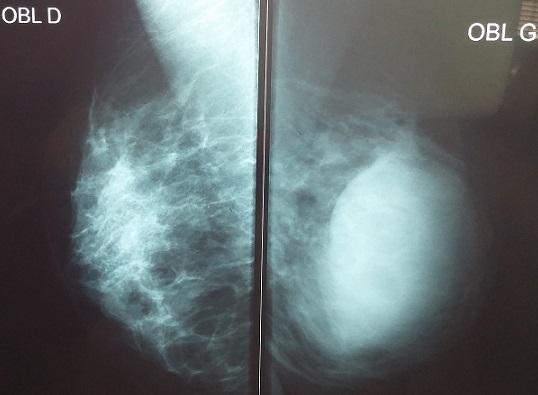
Mammographie de profil: volumineuse masse de contours réguliers de densité moyenne

**Figure 3:**
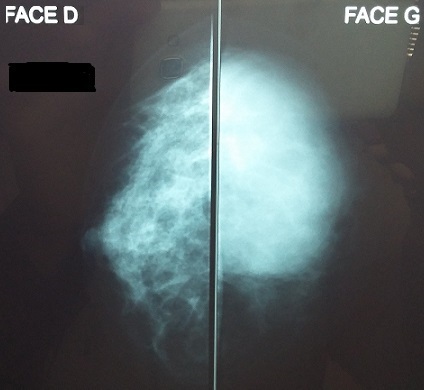
Mammographie de face: volumineuse masse de contours réguliers de densité moyenne

**Figure 4:**
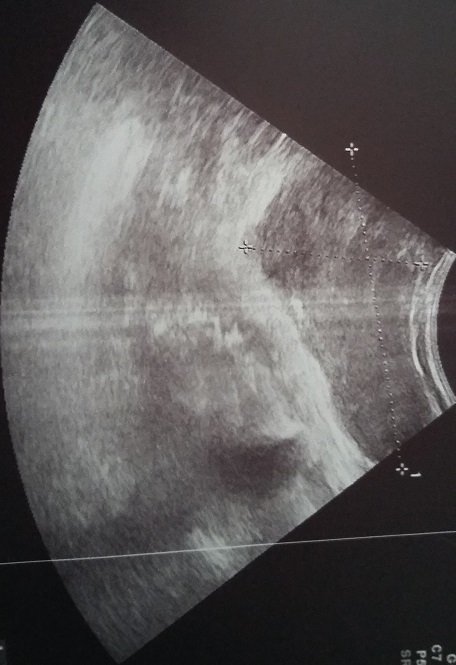
Échographie mammaire: volumineuse collection à contenu échogène

**Figure 5:**
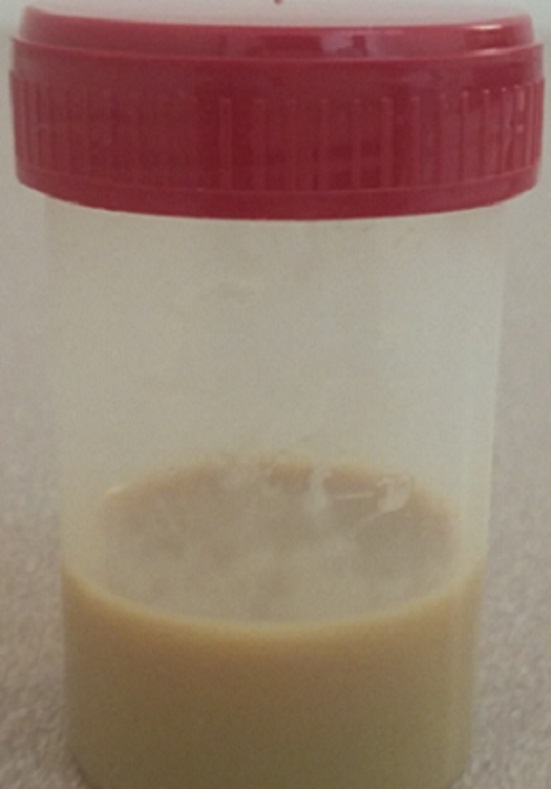
Liquide de la ponction aspiration du galactocèle à l'aiguille fine

Nous rapportons le cas d'une patiente âgée de 30 ans ménarchée à l'âge de 14 ans, G2P2; premier accouchement à l'âge de 16 ans, le second à 19 ans, avec allaitement au sein pendant deux ans pour les deux enfants, divorcée. Le début de sa symptomatologie remontait à l'âge de 21 ans par l'installation d'aménorrhée secondaire et galactorrhée bilatérale. Sur la base de taux élevés de prolactine sérique à 140/ml (plage normale jusqu'à 20 ng/ml) une IRM pelvienne a été demandée laquelle a objectivé: micro adénome hypophysaire latéralisé à gauche de 6 mm de grand axe sans autres anomalies cérébrales associées. Les tests hormonaux et biochimiques n'ont montré aucune autre pathologie qui pourrait induire l'hyperprolactinémie comme l'hypothyroïdie primaire, macroprolactinémie, insuffisance hépatique ou rénale. La patiente fut mise sous Cabergoline*, avec bonne évolution clinique et biologique. Néanmoins, après 8 ans de traitement elle a arrêté son médicament d'elle-même. Vu que la patiente a constaté une augmentation du volume du sein gauche évoluant sur deux mois après une année de sevrage elle a consulté dans notre service. L'examen clinique a objectivé: sein gauche augmenté de taille, masse de consistance molle, prenant tout le sein faisant 12cm/7, mobile par rapport aux deux plans, galactorrhée bilatérale, le sein controlatéral est sans particularités, les aires ganglionnaires sont libres (Figure 1). La mammographie a objective: présence d'une volumineuse masse occupant les quadrants externes du sein gauche, de contours réguliers de densité moyenne mesurant 5.5/6.5/4.5cm (Figure 2, Figure 3). L'échographie mammaire a objectivé: volumineuse collection occupant la quasi-totalité du sein gauche, à paroi fine, à contenu épais et mobile, non vascularisé au doppler semblant communiquer avec des canaux galactophoriques qui sont dilatées, cette collection mesure approximativement 10.3/5.7cm (Figure 4). La patiente a bénéficié d'une ponction aspiration du galactocèle à l'aiguille fine ramenant un liquide visqueux et laiteux mais un peu verdâtre, avec étude cytologique (Figure 5). Le traitement fut redémarré à base de Cabergolide* 0.75 mg/semaine avec bonne évolution clinique, biologique et radiologique.

## Discussion

Les galactocèles sont décrits avec des fréquences différentes en fonction de de l'âge ou de la grossesse des patientes. Par exemple, la prévalence chez les femmes enceintes étudié pour des symptômes mammaires (douleur mammaire, nodules palpables ou de l'échec de la lactation spontanée unilatérale) varie entre 20% [2] et 44% [3]. Dans une étude rétrospective de toutes les biopsies du sein et mastectomie réalisées en 26 ans dans un hôpital d'Arabie Saoudite, de 915 cas, seulement 2.4% avéré être galactocèle. Les lésions les plus fréquentes ont été identifiées fibroadénomes (30.7%), suivie des lésions fibrokystiques (21.1%) et les carcinomes mammaires (14.9%). Les auteurs relient l'incidence élevée de la pathologie associée à la grossesse ou l'allaitement (mastite, abcès, fibroadénomes ou galactocèle) avec le taux élevé de fécondité dans la population [4]. Une autre étude réalisée sur 225 patientes avec différentes plaintes de sein sur une période de 7 ans a identifié le galactocèle en seulement neuf patients (4%) [5]. Sur 715 lésions identifiées chez 8631 les femmes chez lesquelles une échographie a été réalisée, ont été identifiés seulement 33 galactocèle chez 31 patients [6]. Sur une période de 10 ans, chez 1416 patientes ayant une tumeur du sein, seulement 3 galactocèle ont été diagnostiqués [7]. La lésion est considérée comme spécifique chez les jeunes femmes fertiles. Il est découvert pendant ou après l'allaitement ou extrêmement rares, plus tard dans la vie, même après la ménopause. En cas de grossesse multiple, le kyste rechute habituellement. Il y a quelques petites études dans la littérature faisant référence à ces idées. Par exemple, dans une étude britannique réalisée sur 8 femmes avec galactocèle, 5 étaient encore allaitantes et 2 avaient accouché l'année dernière. La lésion de la huitième patiente avait été trouvée dans la même position au cours d'une grossesse précédente [8]. Chez toutes les 11 femmes avec galactocèle identifiées dans une étude espagnole, la masse du sein s'est développée au cours de la période d'allaitement [9]. L'un des rapports les plus curieux se réfère à un galactocèle décrit 2 ans après la ménopause [10] ou encore, 8 parmi 21 cas de galactocèle identifiés dans la première année post ménopause dans un rapport coréen [11]. Une autre étude sur plus de 33 galactocèle découverts après l'allaitement a montré une période d'apparition de 3 à 41 ans après la dernière grossesse [6]. En cas de kystes puerpérales, leur développement est facilité par la vidange incomplète des seins, comme dans la succion inefficace, l'excès de la production du lait ou de modifications dans le programme d'alimentation [8, 12].

Alors que les cas typiques de galactocèle se réfèrent à l'allaitement, il y a des cas extrêmement rares qui ne sont pas liés à l'allaitement, comme notre cas. Un cas a été décrit en rapport avec une obstruction par un papillome intracanalaire [13], et plusieurs cas ont été liés aux prothèses mammaires avec gel de silicone [14, 15]. Dans les cas décrits chez les hommes l'obstacle anatomique est jugé moins important, que les changements hormonaux. Chez les mâles nouveau-nés, les galactocèles constituent une complication de la crise génitale néonatale, apparaissant généralement de moins de 1 an [16–18] ou très rarement plus tard dans la vie [19]. Dans un cas, la lésion était présente chez un enfant avec hypopituitarisme congénitale [20]. La prolactine agit comme un stimulant pour le sein. Il y a un rapport sur des patients (hommes) de 19 ans, avec des niveaux extrêmement élevés de la prolactinémie avec un macroprolactinome ayant un fluide 50 ml galactocèle. Le pronostic à long terme du galactocèle semble être bon. Par exemple, sur 5 patients qui ont développé la lésion pendant la grossesse ont été suivis pendant 27 mois et aucun développé un carcinome mammaire [2]. Le tableau clinique du kyste n'est pas différent des autres kystes mammaires avec différentes étiologies. Les galactocèle apparaissent sous forme de nodules uniques ou multiples qui se développement au cours des semaines ou des mois, mais ils peuvent continuer d'augmenter de taille pendant des années [10]. La lésion est le plus souvent localisée dans le quadrant supéro-externe, la lésion occupait la quasi-totalité du sein gauche du sein dans notre cas. Les diamètres moyens sont de 1 jusqu'à 6cm, et le diamètre maximal décrit était de 14cm. Notre patient avait un kyste qui a grandi suite à l''arrêt du traitement mesurant 10.3/5.7cm. En général, les kystes sont indolores, bien circonscrits et mobiles. Les signes inflammatoires sont absents. Très rarement, en raison de la croissance rapide, la douleur locale peut être présente. Notre jeune patiente avait une légère mastodynie gauche; avec une masse molle, mobile de 12/7cm seul examen physique délectait asymétrie du sein. Les examens radiologiques comprennent principalement le balayage ultrasonore et la mammographie. Sur le premier, l'image classique est d'un kyste bien limité, avec une paroi fine, le contenu est échogène ou hypoéchogène ou mixte. Il peut y avoir des lésions uniques ou multiples, généralement de forme ovalaire. Cet aspect est rencontré dans environ trois quarts des cas [8] même dans notre cas. Les kystes sont généralement petits, mais les formes géantes jusqu'à 14cm ont été décrites [6, 8]. Parfois, on a des formes atypiques de galactocèle mimant une masse solide, l'aspect hypoéchogène peut être homogène ou avec des nodules à l'intérieur. Cette image est peut être causée par la réflectivité élevée de la graisse dans le galactocèle. Parfois, la lésion nest pas bien limitée ou on peut avoir des marges irrégulières, soulevant la possibilité d'un carcinome intra-kystique associé. Il semble que l'aspect classique est plus fréquemment rencontré dans la première année après une grossesse, alors que les galactocèle diagnostiqués plus tard sont généralement solides, probablement en raison de la fibrose et de la réorganisation du tissu [11]. L'image de notre cas est typique malgré qu'il ne soit pas lié à l'allaitement.

Les dispositifs de mammographie se réfèrent au galactocèle comme étant bien limité, une lésion unique, ou multiple avec une densité égale ou inférieure à celle du reste du sein, comme dans le niveau de liquide. Cet aspect apparaît sur l'incidence latérale et peut être absent si le cliché est oblique (incidence qui est préféré, car il comprend l'extension axillaire du sein). Parfois, la lésion présente des zones de différentes densités mélangées ensemble, comme dans un hamartome. En raison de la résorption du fluide, un galactocèle ancien apparait complètement transparent. Parfois, la lésion peut avoir des calcifications. Le liquide trouvé dans les kystes peut être blanc et fluide si frais ou ayant une viscosité plus élevée si la lésion est plus ancienne. Le fluide contient des protéines, de matières grasses et de lactose dans des proportions différentes. Un test mucique identifie le galactose et confirme le diagnostic de lait [7]. Les cellules sont représentées par de rares cellules mousseuses et des cellules de l'épithélium bénin avec des changements de lactation. Les caractéristiques histopathologique proviennent du fait que le galactocèle est une dilatation des conduits avec du lait produit par les acines environnantes. Le kyste est bordée par un épithélium canalaire avec des changements de lactation (cellules cuboïdes avec vacuoles sécrétrices contenant des lipides) [8, 20]. En raison de la pression provenant du fluide, l'épithélium présente des zones de nécrose et de l'inflammation chronique ou aiguë stérile. Autour du kyste, il y a des glandes apocrines actifs. Le diagnostic différentiel de graisse contenant des lésions kystiques du sein comprend les lipomes ou des kystes résultant de la résorption d'hématomes. Les lésions de densité mixte doivent être différenciées des ganglions lymphatiques intra mammaires ou fibroadeno-lipomes ou hamartomes. Les carcinomes mammaires peuvent parfois avoir un aspect piège et peuvent imiter une telle image. Le traitement classique et également la meilleure man'uvre de diagnostic pour un galactocèle se compose d'aspiration par aiguille fine. Le liquide laiteux obtenu confirme le diagnostic. Les incidents possibles de cette méthode sont la douleur locale, une infection ou la récidive. Quoi qu'il en soit, les galactocèles ont tendance à croître à nouveau, quelques fois au cours d'une grossesse ultérieure. Dans ce cas, l'aspiration peut être utilisé à nouveau, ou le kyste peut être enlevée chirurgicalement avec le conduit obstrué, mais la chirurgie prophylactique est généralement pas recommandé [8, 12]. La prévention des kystes peut être tentée chez les patients post-partum, en vidant complètement le sein à chaque tétée. Masser les seins, des douches chaudes pour faciliter la libération du lait. Chez les patients qui ont développé un galactocèle au cours d'une grossesse le kyste peut réapparaître. La chirurgie préventive est généralement pas recommandée, même pas dans le cas de grandes galactocèle.

## Conclusion

Contrairement à la majorité des cas décrits dans la littérature, notre cas n'a pas fait référence à une période de grossesse ou d'allaitement. L'hyperprolactinémie semble être la seule cause. Mais la bonne évolution sous traitement a été concluante pour le diagnostic. En conclusion, nous soulevons la question d'une corrélation entre les taux sériques élevés de prolactine et sa cible mammaire qui peut induire une galactocèle mais d'autres études sont nécessaires pour confirmer l'hypothèse.

## Conflits d’intérêts

Les auteurs ne déclarent aucun conflit d'intérêts.
